# The effects of ear acupressure therapy on obstetric and gynecological pain in women: a systematic review and meta-analysis

**DOI:** 10.7586/jkbns.24.035

**Published:** 2024-11-26

**Authors:** Mi-Kyoung Cho, Mi Young Kim

**Affiliations:** 1 Department of Nursing Science, Research Institute of Nursing Science, Chungbuk National University, Cheongju, Korea; 2 College of Nursing, Hanyang University, Seoul, Korea

**Keywords:** Acupressure, Gynecology, Meta-analysis, Pain, Systematic review

## Abstract

**Purpose:**

This study aimed to evaluate the effectiveness of ear acupressure therapy on obstetric and gynecological (OBGY) pain in women through a systematic review and meta-analysis, providing foundational data on complementary and alternative therapies.

**Methods:**

Using the PICO-SD framework, searches were conducted across seven electronic databases: PubMed, Cochrane, EMBASE-OVID, CINAHL, Web of Science, Scopus, and RISS. The population (P) was women; the intervention (I) included ear acupressure, acupuncture, or auriculotherapy; the control (C) was usual obstetric care or placebo therapy; the outcome (O) was pain; and the study design (SD) was a randomized controlled trial (RCT). Study quality was assessed using the Joanna Briggs Institute Revised Checklist for RCTs. Meta-regression and exclusion sensitivity analyses were also performed.

**Results:**

Sixteen studies were included, with a pooled overall effect size of Hedge’s g = −1.41 (95% confidence interval: −1.96 to −0.86). Meta-regression revealed that factors such as publication year, region, institutional review board approval, funding, sample size, type of pain, intervention type, tools, stimulus points, control group type, intervention duration, session count, session time, outcome measurement time, and quality assessment scores significantly influenced pain reduction.

**Conclusion:**

The findings confirm that ear acupressure therapy effectively reduces OBGY pain. However, given the variations in application methods and resulting differences in effectiveness, it is necessary to carefully consider factors such as tools, stimulus points, intervention methods, control group interventions, duration, session count, and session time when applying the therapy.

## INTRODUCTION

Women experience significant pain related to obstetric and gynecological (OBGY) issues. Among these, childbirth is a crucial experience for women, and the pain and anxiety encountered during labor can be extremely challenging [1]. In addition to this, there are various types of pain, including premenstrual syndrome symptoms, pain related to breastfeeding, and pain from cesarean sections. Although pharmacological analgesia is commonly used to relieve labor pain, concerns arise due to its side effects and invasive nature. Furthermore, even after the administration of medication, women may continue to experience pain [2]. Additionally, medications like non-steroidal anti-inflammatory drugs are associated with various side effects, including gastrointestinal issues, nephrotoxicity, blood disorders, headaches, and drowsiness [3]. As a non-pharmacological approach, complementary and alternative therapies can also be utilized. The mechanism of pain involves the detection of harmful stimuli in the peripheral nervous system, which are then transmitted to the central nervous system. These neural signals are recognized and interpreted in the brain, leading to the subjective experience of pain [4]. Considering these characteristics, it is necessary to explore effective methods for pain relief.

As one of these methods, Auricular point therapy, a form of acupuncture, can be considered as a complementary and alternative therapy. This therapy has been gaining popularity as a non-invasive and effective method for pain management. It involves stimulating specific points on the ear using various techniques, such as needle embedding, ear pressure probes, and auricular point sticking. Auricular acupuncture is a combination of traditional Chinese and Western medicine, widely applied to treat headaches, chest pain, dysmenorrhea, lower back pain, and skin and muscle pain [5].

The mechanism of auricular point therapy is understood as follows: The pain-relieving effect appears almost immediately after pressing on the auricular points. According to the meridian theory of Traditional Chinese Medicine, the auricles are directly or indirectly connected to the 12 meridians that traverse the entire body, involving visceral and endocrine-related areas innervated by the extremities and the vagus nerve. Specific points, such as the Internal Genitalia point, correspond to the functions and organs of the upper reproductive tract, including the hypothalamus-pituitary-ovary axis [6]. Similarly, the Shenmen point corresponds to the functions of the vagus nerve system [7]. In modern medicine, the neurohumoral theory suggests that stimuli such as acupuncture or electroacupuncture regulate and integrate the neuroendocrine-immune network system within the body [8]. Previous studies have shown inconsistent or conflicting results regarding the effects, indicating a need for further research. Although auriculotherapy is known to be effective, a systematic review is needed to determine whether it significantly reduces OBGY pain in women. Furthermore, since auriculotherapy can be applied in various ways, it is essential to evaluate which method is the most effective. The areas of the ear used for treatment vary, as do the number of applications. For example, participants in the intervention group are instructed to press the seeds six times a day, 20 minutes before each meal [9]. Pain varies in threshold among individuals, is subjective, and has diverse mechanisms; however, the Visual Analog Scale, which allows for intuitive measurement, is commonly used for quantitative assessment. There are also several types of ear acupressure tools. Techniques for auricular acupressure include electrical stimulation, needles, lasers, cupping, or pellet labels (containing magnetic or plant-based particles like Vaccaria) and passive pressure on the ear [10]. Given the variability in the principles and application methods of auriculotherapy, it is necessary to conduct a systematic study to identify which technique is the most effective for managing OBGY pain in women.

Therefore, this study aims to investigate the effects of complementary and alternative therapies through a systematic review and meta-analysis, focusing on the impact of ear acupressure therapy on OBGY pain in women.

## METHODS

### 1. Inclusion and exclusion criteria

In this study, the selection criteria were based on the PICO-SD framework (Population, Intervention, Comparison, Outcome, and Study Design) [11]. The population (P) was women; the intervention (I) included ear acupressure, acupuncture, or auriculotherapy; the control group (C) consisted of usual obstetric care or placebo therapy; the outcome (O) was pain; and the study design (SD) was randomized controlled trials (RCTs).

The inclusion criteria consisted of studies reported in either Korean or English, with at least two measurements taken before and after the intervention. For effect size calculation, the first measurement taken at the endpoint of the intervention, rather than intermediate measurements during the intervention, was used. Only studies that provided sample size, mean, and standard deviation in the results were selected to ensure accurate effect size calculations.

The exclusion criteria were studies involving participants under the age of 19, studies where the intervention site was not the ear, studies that did not report pain as an outcome variable, studies not published in Korean or English, studies where the full text was unavailable, and single-group studies without a control group.

### 2. Search strategy and data sources

The literature search was conducted across seven electronic databases: PubMed, Cochrane, EMBASE-OVID, CINAHL, Web of Science, Scopus, and RISS between June 19, 2024, and July 19, 2024. The search terms included MeSH terms such as ‘women,’ ‘Acupuncture, Ear,’ and ‘pain,’ along with relevant natural language terms such as ‘ear pressing’, ‘auricular pressing’, ‘auriculoacupressure’. Filters were applied to limit results to studies involving humans, published in Korean or English, RCTs, and full-text available articles published in each database, either online or offline, by July 31, 2024. The search was performed by a librarian and one researcher, and another researcher cross-checked the search results.

The meta-analysis for this study followed the Preferred Reporting Items for Systematic Reviews and Meta-Analyses (PRISMA) 2020 checklist(accessed on June 30, 2024, from https://prisma-statement.org/PRISMAStatement/Checklist.aspx). The search protocol was registered on PROSPERO, the International Prospective Register of Systematic Reviews (registration No. CRD42024598617), on October 18, 2024 (https://www.crd.york.ac.uk/prospero).

### 3. Data extraction

The bibliographic data of selected studies were organized in Excel, with each study assigned a serial number and consolidated in a sheet by author, publication year, title, and journal. Duplicate records were filtered by title and author. Titles, abstracts, and full texts were reviewed to select studies based on inclusion and exclusion criteria, with exclusion reasons noted (e.g., language, target population, study design, and statistical data). Consistency was ensured through cross-checking at each screening stage.

The selected studies were organized in Excel, with each study assigned a serial number and documented by author, publication year, title, and journal. Duplicates were filtered by title and author. Titles, abstracts, and full texts were reviewed based on inclusion and exclusion criteria, with exclusion reasons (e.g., language, target population, study design) recorded and cross-checked for consistency.

Eligible studies were documented in a coding book, detailing authorship, publication year, institutional review board (IRB) approval, funding status, participant characteristics (sample size, group distribution, pain type), research design, and intervention specifics (e.g., stimulus type, ear acupressure tool, acupoint locations, control intervention, session details, outcome measures, and quality scores).

In the meta-analysis, the experimental group consisted of participants receiving ear acupressure, and the control group included those receiving no intervention or standard care. Sample sizes were calculated by summing participants across groups. Data were independently coded by two researchers, with discrepancies resolved through review and consensus. For meta-analysis, the experimental group included those receiving ear acupressure, and control groups receiving no intervention or standard care. Sample size was calculated by summing participants from both groups. Data were independently coded by two researchers, with discrepancies resolved through review and consensus.

### 4. Quality assessment

The quality of the selected studies was assessed using the Revised Checklist for Randomized Controlled Trials from the Joanna Briggs Institute (JBI) [12]. The JBI Checklist for Randomized Controlled Trials consists of 13 items, divided into two categories: risk of bias to assess internal validity (10 items) and statistical conclusion validity(3 items). Each item scored 1 point for ‘clear,’ and 0 points for ‘unclear,’ ‘no,’ or ‘not applicable.’

In this study, all 16 studies included in the analysis were RCTs, with a mean quality score of 10.19 ± 1.72. Among the items, ‘Q5. Were those delivering the treatment blind to treatment assignment?’ was not clearly reported in any of the studies. ‘Q4. Were participants blind to treatment assignment?’ was reported clearly in seven studies (Study ID: 3, 4, 5, 6, 7, 9, 10). Similarly, ‘Q2. Was allocation to treatment groups concealed?’ was reported clearly in nine studies (Study ID: 3, 4, 5, 6, 7, 9, 10, 11, 14), as was ‘Q7. Were outcome assessors blind to treatment assignment?’ (Study ID: 1, 2, 5, 6, 7, 8, 9, 10, 14). The detailed results are presented in Appendix 1, 2.

### 5. Statistical analysis

The overall effect size of the studies included in the analysis was calculated, and the validity of the meta-analysis was evaluated using MIX 2.0 Pro Ver. 2.0.1.6 (BiostatXL) [13]. The effect sizes of individual studies and the pooled overall effect size are expressed as Hedge’s g. Hedges's g is a statistical measure used to assess the effect size in inferential statistics. It is typically calculated using the square root of the mean square error derived from an analysis of variance that tests for differences between two groups. The interval estimates were calculated as 95% confidence intervals (CI), and the weights for each effect size were derived using the inverse of variance [14]. The pooled overall effect size is estimated using a random effects model, which adjusts the weights to account for both within-study variability and between-study heterogeneity [15].

The heterogeneity among the included studies was assessed using Cochrane’s Q-statistics and Higgins' I^2^, which represents the proportion of variance across studies due to heterogeneity [16]. A *p*-value of less than 0.05 in the Q-statistics or an I^2^ value exceeding 50% was interpreted as indicating significant heterogeneity [17]. To identify the factors contributing to heterogeneity in studies on OBGY pain, subgroup analysis, meta-regression, and exclusion sensitivity analyses were conducted [18]. Meta-regression analysis is a statistical technique that involves the regression of summary estimates from individual studies on one or more covariates. In this study, we used a fixed-effect linear meta-regression model, predicated on the assumption that all heterogeneity between studies could be adequately explained by the covariates included in the regression model [19]. Additionally, publication bias was assessed using funnel plots, trim-and-fill plots, Begg’s test, Egger’s regression, and the trim-and-fill method to adjust and validate the overall effect [20].

## RESULTS

### 1. Characteristics of the included studies

In this study, the selection of target studies for analysis followed a three-step process in accordance with the PRISMA guidelines, as depicted in Appendix 3. Among the 16 studies included, 11 were published in Asia and 11 were published after 2019. A total of 15 studies had obtained IRB approval, and 11 studies had received funding. All studies employed a RCT design, and five studies included more than 100 participants. Regarding the types of pain addressed, seven studies focused on labor pain during normal delivery, four studies on cesarean section, two studies on surgical procedures such as episiotomy, and three studies on other types of breastfeeding pain, pregnancy-related back pain, and premenstrual symptoms.

In terms of intervention methods, 13 studies used acupressure, while three studies used acupuncture. Regarding intervention tools, seven studies utilized seeds, and three studies used needles. Ten studies applied fewer than five stimulus points to reduce pain, and four studies used alternative or placebo interventions for the control group. The duration of the intervention was more than two days in six studies, and six studies included more than two sessions. The intervention time per session was more than 75 seconds in four studies. For outcome measurement, two studies evaluated the long-term effect of the intervention. The quality assessment scores were 10 or higher in 11 studies (Table 1, 2).

### 2. The effect of ear acupressure therapy on OBGY pain

**Figure 1.**
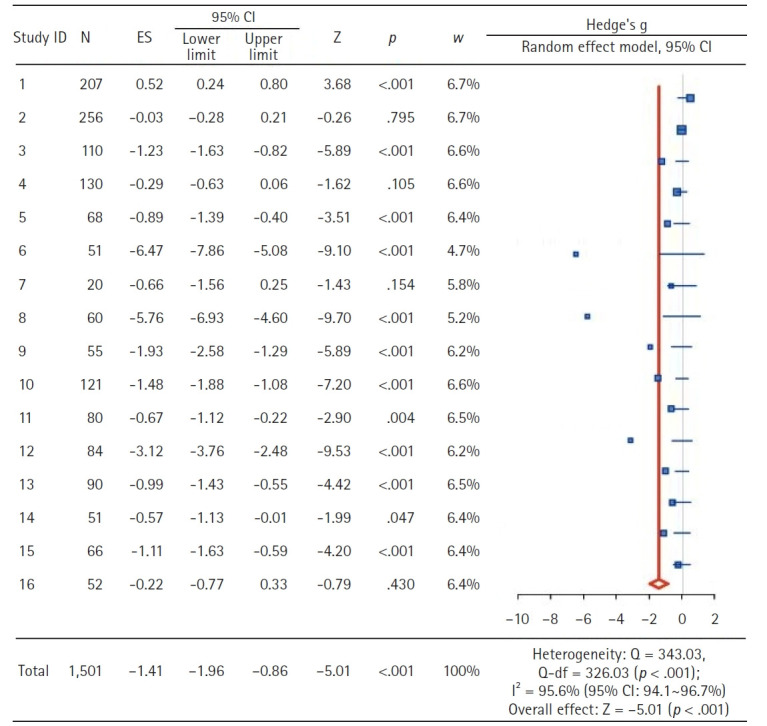
The effects of ear acupressure intervention on obsteric and gynecological pain. N = Number; ES = Effect size; CI = Confidence interval.

The pooled overall effect size of the 16 studies on ear acupressure therapy for OBGY pain was Hedge’s g = −1.41 (95% CI: −1.96 to −0.86). According to Brydges' [21] criteria for interpreting effect sizes, this result indicates a large effect (Figure 1). In the heterogeneity test, the Q-statistics was 343.03 (*p* < .001), and Higgins' I^2^ was 95.6%, indicating high heterogeneity among the studies. To explore the sources of heterogeneity, subgroup analysis, and meta-regression analysis were conducted.

The subgroup analysis revealed that studies published after 2019 (Hedge’s g = −1.70, 95% CI: −2.34 to −1.06) and those conducted in Asia (Hedge’s g = −1.38, 95% CI: −1.99 to −0.77) demonstrated statistically significant pain reduction effects. Studies with fewer than 100 participants (Hedge’s g = −1.90, 95% CI: −2.68 to −1.13) also showed significant results. The type of pain was another influential factor, with normal delivery (Hedge’s g = −1.92, 95% CI: −2.93 to −0.92) and cesarean section (Hedge’s g = −0.96, 95% CI: −1.51 to −0.40) yielding different effect sizes. Intervention methods also played a role; acupressure as the intervention type resulted in Hedge’s g = −1.28 (95% CI: −1.81 to −0.75). When needles were used as the ear acupressure tool, the effect size was Hedge’s g = −0.44 (95% CI: −0.81 to −0.08). Studies using no intervention or routine obstetric care as the control group intervention achieved Hedge’s g = −1.86 (95% CI: −2.52 to −1.20). Additionally, studies with session durations of less than 75 seconds or not reported showed Hedge’s g = −1.56 (95% CI: −2.18 to −0.94) (Table 2).

A univariate meta-regression was performed to assess the potential impact of study characteristics on effect size. The results indicated that studies published after 2019 (Z = −10.29, *p* < .001) and those conducted in Asia (Z = 3.11, *p* = .002) had statistically significant effects. The absence of IRB approval (Z = 10.52, *p* < .001) and funding (Z = 3.73, *p* < .001) were also associated with significant findings. Studies with fewer than 100 participants (Z = 8.54, *p* < .001) and those targeting pain types such as normal delivery (Z = −6.81, *p* < .001), cesarean section (Z = −2.83, *p* = .005), Episiotomy (Z = 11.29, *p* < .001) and other pain conditions(Z = −3.91, *p* < .001) exhibited significant influences. Acupressure as the intervention type (Z = −3.90, *p* < .001) and the use of tools other than needles or seeds, such as beads or magnetic plates (Z = −2.46, *p* = .014), were also significant factors. Fewer than five stimulus points (Z = −10.07, *p* < .001) and control groups receiving no intervention or routine obstetric care (Z = 10.68, *p* < .001) further contributed to the results. Intervention durations exceeding two days (Z = 2.23, *p* = .026) and fewer than two sessions or unreported session numbers (Z = −8.29, *p* < .001) were also influential. Longer session times exceeding 75 seconds (Z = 4.64, *p* < .001) and outcome measurements not conducted immediately after the intervention (Z = −11.03, *p* < .001) impacted the findings. Finally, studies with quality assessment scores of 10 or higher (Z = 4.27, *p* < .001) demonstrated statistically significant effects (Table 3).

The exclusion sensitivity test [22] evaluated the combined effect size and statistical significance after reducing weights. The Hedge’s g ranged from −1.54 to −1.15, indicating a large effect size, with a 95% CI between −2.13 to −1.66 and −0.99 to −0.64, demonstrating statistically significant sensitivity to exclusion (Appendix 4).

### 3. Publication bias

**Figure 2.**
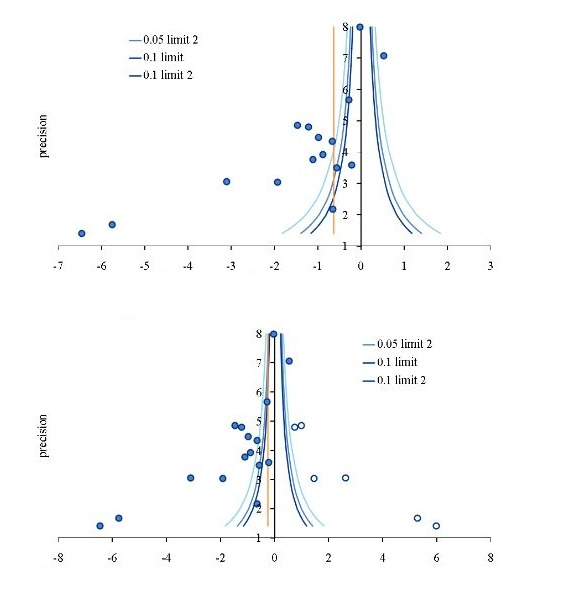
(A) Funnel plot of ear acupressure intervention on obsteric and gynecological pain. (B) Trim and fill plot of ear acupressure intervention on obsteric and gynecological pain. Precision = 1/standard error; 0.05; limit line = 95% confidecne limit.

To assess publication bias, funnel plot, and trim-and-fill plot analyses were performed. The individual effect sizes of the 16 studies included in this research (blue circles) appeared skewed to the left, indicating the presence of publication bias (Figure 2-A). The trim-and-fill plot suggested that six additional studies (white circles) should be added (Figure 2-B).

Further analysis using the trim-and-fill method [23] confirmed that six studies were missing, and the adjusted pooled effect size of the 22 studies was −0.25 (95% CI: −0.35 to −0.15). Even after adjusting for publication bias, the effect size for pain reduction remained statistically significant.

Additionally, both Begg's test for rank correlation and Egger's regression test showed significant results for pain reduction, confirming that while publication bias was present, it did not alter the statistical significance of the results. Therefore, the findings were deemed acceptable despite the presence of publication bias (Appendix 5).

## DISCUSSION

This study was conducted to determine the effects of ear acupressure therapy on OBGY pain in women. The results showed that the pooled overall effect size for ear acupressure therapy on OBGY pain across the 16 studies was Hedge’s g = −1.41 (95% CI: −1.96 to −0.86). According to Brydges' [21] criteria for interpreting effect sizes, this indicates a large effect. These findings align with previous studies demonstrating the effectiveness of ear acupressure in reducing pain during childbirth [24] and in managing postoperative pain following cesarean section [25]. OBGY pain, particularly labor pain, is one of the most intense types of pain, significantly affecting the psychological and emotional well-being of pregnant women [26]. Moreover, women respond differently to labor pain, which adds to the complexity of pain management [27]. The results of this study also support previous research suggesting that ear acupressure is effective for managing symptoms of premenstrual syndrome [28].

Furthermore, ear acupressure is easy, safe, and can be self-administered, making it a practical intervention not only for healthcare providers but also for individuals themselves. Given these advantages, its use in combination with other methods for treating obesity could become widely applicable in clinical practice. A univariate meta-regression was conducted to assess the potential impact of study characteristics on effect size. The analysis identified several significant factors influencing OBGY pain in women, including publication year, publication region, IRB approval, funding status, sample size, type of pain, intervention type, intervention tool, number of stimulus points, control group type, number of intervention sessions, intervention time per session, outcome measurement time, and quality assessment score. The high heterogeneity observed in this study is thought to be due to the wide range of situations in which obstetric pain occurs, leading to diverse pain characteristics and varied methods of pain relief. Therefore, caution is needed when interpreting the results due to this high heterogeneity. Publication bias is common and can lead to an overestimation of effect size; therefore, caution is needed when interpreting the results.

A closer examination revealed that studies published after 2019, those conducted in Asia, studies without IRB approval or funding, and those with fewer than 100 participants had a statistically significant effect on the reduction of OBGY pain. However, further research is required to explore these findings in greater depth, as the current evidence is insufficient to draw definitive conclusions.

The results also indicated that the type of pain and intervention method—whether normal delivery, cesarean section, or other types of pain—were statistically significant factors. This finding suggests that the effectiveness of interventions varies according to the type of pain. Specifically, it aligns with previous research indicating that auricular acupressure helps alleviate the pain and anxiety experienced by women in the latent phase of labor and that these pain-relief effects manifest rapidly as uterine contractions increase [23].

The intervention tools used in the studies included various instruments such as magnetic beads, needles, Vaccaria seeds, and other types of seeds. Among these, the use of beads or magnetic plates, rather than needles or seeds, was found to be more effective in reducing OBGY pain in women. Different tools can stimulate these points, including seeds, needles, lasers, or electric stimulation. In particular, Vaccaria seeds are applied using patches, allowing the patient to perform the stimulation independently without needing further evaluation by an operator [29]. Furthermore, as noted in previous research, the most commonly used materials in auriculotherapy are semipermanent needles and mustard seeds [30]. The findings of this study suggest that the effectiveness of auriculotherapy may vary depending on the type of tool used, with beads or magnetic plates showing significant efficacy in reducing OBGY pain in women.

The stimulus points also varied in location and number. The results showed that studies using fewer than five stimulus points were more effective in reducing OBGY pain. The principle of auriculotherapy involves reducing prostaglandin levels, regulating nitric oxide, increasing β-endorphin levels, blocking calcium channels, and improving uterine circulation through uterine pathways to alleviate pain [31]. However, the findings suggest that the number of stimulus points and the appropriate stimulation may not be directly related to pain reduction outcomes.

Additionally, significant pain relief was observed when the control group received no intervention or routine obstetric care, when the intervention duration was more than two days when the number of sessions was fewer than two or not reported, and when the intervention time per session exceeded 75 seconds. In addition, pain reduction was more effective when the outcome measurement time was not conducted immediately after the intervention. This suggests that the intervention has a delayed effect, indicating not only temporary and immediate relief but also the potential for sustained effects. Further research is needed to explore this finding in more detail.

The results also showed that studies with quality assessment scores of 10 or higher had a statistically significant impact on pain reduction. This finding implies that well-designed studies tend to produce more effective results, underscoring the need for rigorous study design to accurately identify the effectiveness of interventions.

The limitations of this study are as follows. Obstetric pain is diverse, but most studies included in this research focus on pain related to childbirth, surgical pain, and episiotomy, with only a few addressing menstrual pain. This raises concerns about heterogeneity and publication bias, which may distort the potential effect size. Therefore, caution is needed when generalizing these results.

## CONCLUSION

In conclusion, the findings of this study demonstrate that ear acupressure therapy is effective in reducing OBGY pain in women. However, given the considerable variation in the application methods of ear acupressure therapy and the resulting differences in effectiveness, it is important to carefully consider factors such as the therapy tool, stimulus points, intervention method, control group intervention, intervention duration, number of sessions, and intervention time per session when applying the intervention. Furthermore, considering the high heterogeneity observed in this study, future research is recommended to secure a more diverse range of studies to further investigate the intervention's effectiveness.

## Figures and Tables

**Table 1.** Descriptive Summary of the Included Studies

Study ID	Author (yr)	Country	IRB	Fund	Participants	Research design	Stimulus type	Acupressure tool	EG intervention (ear acupoints)	CG intervention	Program duration	Program session	Session time	Measurement time (post-test)	Outcome variables (scale)
1	Kindberg et al. (2009)	Denmark	Yes	Yes	207 primiparous women with vaginal delivery who needed surgical repair (E: 105, C: 102)	RCT	Acupuncture	Needles	Four acupoints (genital, Shen men, bladder 36, governor vessel 20)	Local anesthetics	1 day	1	A few minutes	Immediately	- Pain (VDPS, VAS)
															- Wound healing (REEDA scale)
2	Kwan & Li (2014)	Hongkong	Yes	Yes	256 women during the first 48 hours after delivery (E: 126, C: 130)	RCT	Acupressure	Vaccaria seeds	Four acupoints (auricle, Shen men, external genital, anus)	Placebo	2 days	1	30 sec	Immediately	- Pain (VDPS, VAS)
															- Paracetamol consumption
3	Vas et al. (2019)	Spain	Yes	Yes	110 pregnant women diagnosed with pregnancy-related lower back and posterior pelvic girdle pain (E: 55, C: 55)	RCT	Acupuncture	Needles	Three acupoints (Shen men, kidney, lumbar or sacral regions)	Routine obstetric care	2 weeks	2	Not reported	Immediately	- Pain (VAS)
															- RMDQ
															- Health status (SF-12, short form-12 health survey)
4	Rakchanok et al. (2022)	Thailand	Yes	Yes	130 primigravida pregnant women who underwent cesarean delivery (E: 65, C: 65)	RCT	Acupressure	Magnetic plates	Three acupoints (Shen men, Erzhong, Penqiang)	Routine obstetric care	3 days	None reported	1-2 min	Immediately	- Pain (VAS)
5	Mafeton et al. (2018)	Brazil	Yes	No	68 parturients w/ dilation ≥ 4 cm (E: 34, C: 34)	RCT	Acupressure	Crystal microspheres	Four acupoints (Shen men, neurasthenia, endocrine, uterus).	Routine obstetric care	1 day	1	1 min	Immediately	- Pain (VAS)
6	Zhu et al. (2023)	China	No	Yes	51 primiparas during the latent period of the first stage of labor (E: 25, C: 26)	RCT	Acupressure	Vaccaria seeds	Four acupoints (internal genitals, Shen men, Jiaowozhong, endocrine)	Routine obstetric care	1 day	1	30 sec	Immediately	- Pain & anxiety (VAS)
															- Montevideo units for uterine contractions
7	Mafetoni & Shimo (2016)	Brazil	Yes	No	20 pregnant women w/ dilation ≥ 4 cm (E: 10, C: 10)	RCT	Acupressure	Crystal beads	Four acupoints (Shen men, uterus, neurasthenia, endocrine)	Routine obstetric care	1 day	1	1 min	Immediately	- Pain (VAS)
8	Korelo et al. (2022)	Brazil	Yes	No	60 women w/ premenstrual syndrome symptoms (E: 30, C: 30)	RCT	Acupuncture	Needles	Seven acupoints (anxiety, endocrine, muscle relaxation, kidney, Shen men, sympathetic)	No intervention	8 weeks	8	Not reported	Delayed	- Severity of PMS symptoms (PSST)
															- Musculoskeletal pain (Nordic questionnaire)
															- Anxiety severity (BAI)
															- QoL (WHOQOL-Bref)
9	Alimoradi et al. (2020)	Iran	Yes	Yes	55 pregnant women (37-42 weeks) (E: 28, C: 27)	RCT	Acupressure	Vaccaria seeds	Seven acupoints (zero, genitalia, Shen men, thalamic, prostaglandin, oxytocin, uterus)	Routine obstetric care	1 day	1	30 sec	Immediately	- Pain (VAS)
10	Marznaki et al. (2023)	Iran	Yes	Yes	121 pregnant women who undersent cesarean section (E: 60, C: 61)	RCT	Acupressure	Finger (thumb & fore)	Six acupoints (Shen man, zero, pelvic, abdomen, endocrine, uterus)	Routine obstetric care	1 day	3	1 min	Immediately	- Pain (VAS)
11	Abedi et al. (2017)	Iran	Yes	No	80 nulliparous women with labor pain (E: 40, C: 40)	RCT	Acupressure	None reported	Eight acupoints (zero, Shen men, uterus, pelvic, abdomen, spleen, external genitalia, master cerebral)	Routine obstetric care	1 day	3	Not reported	Immediately	- Pain (VAS)
12	Valiani et al. (2018)	Iran	No	Yes	84 nulliparous women with labor pain (E: 42, C: 42)	RCT	Acupressure	Seeds	Twelve acupoints (Shen men, zero, thalamus, endocrine, autonomous, cerebral, sensorial, pelvic, uterus, posterior pituitary, prostaglandin, external genitalia)	Routine obstetric care	1 day	2	20 min	Delayed	- Pain (McGil's short-form standard questionnaire, VAS)
13	Maryam et al. (2020)	Iran	Yes	No	90 pregnant women who underwent cesarean section (E: 45, C: 45)	RCT	Acupressure	Seeds	Two acupoints (shoulder, muscle relaxation)	Placebo	2 days	None reported	Not reported	Immediately	- Pain (NRS)
14	Hu et al. (2024)	China	Yes	Yes	51 primiparas w/ 2-3 cm dilation (E1: 25, C: 26)	RCT	Acupressure	Seeds (small blank seeds)	Four acupoints (E3)	Routine obstetric care	1 day	1	30 sec	Immediately	- Labor pain (VAS)
															- Labor anxiety (VAS)
															- Uterine contraction (fetal heart rate monitor)
15	Mousavi et al. (2023)	Iran	Yes	Yes	66 pregnant women for cesarean section (E: 33, C: 33)	RCT	Acupressure	Manual pointer	Five acupoints (Shen men, subcortex, uterus, pelvic, abdominal)	Routine obstetric care	1 day	1	20 min	Immediately	- Anxiety (STAI)
															- Pain (VAS)
16	Han et al. (2024)	Korea	Yes	Yes	52 breastfeeding mothers (E: 26, C: 26)	RCT	Acupressure	Vaccaria seeds	Four acupoints (Shen men, central rim, breast, endocrine)	Placebo	4 weeks	4	Not reported	Immediately	- Breast pain (NRS)
															- Pressure pain thresholds (algometer)

E, EG = Experimental group; C, CG = Control group; IRB = Institutional review board; RCT = Randomized controlled trials; VDPS = Verbal descriptive pain scale; VAS = Visual analog scale, REEDA = Redness, edema, ecchymosis, discharge, approximation; RMDQ = Roland-Morris disability questionnaire; PMS = Premenstrual symptoms; PSST = Premenstrual symptoms screening tool; BAI = Beck anxiety inventory; WHOQOL-Bref = World Health Organization quality-of-life scale; NRS = Numerical rating scale; STAI = The state-trait anxiety inventory.

**Table 2.** Subgroup Analysis Regarding Ear Acupressure on Obstetric and Gynecological Pain by Study Characteristics

Characteristics	Categories	K	Study ID	N	ES	95% CI		Z	*p*
						Lower limit	Upper limit		
Year	< 2019	5	1, 2, 7, 11, 12	647	−0.76	−1.71	0.19	−1.57	.116
	≥ 2019	11	3, 4, 5, 6, 8, 9, 10, 13, 14, 15, 16	854	−1.70	−2.34	−1.06	−5.24	< .001
Country	Asia	11	2, 4, 6, 9, 10, 11, 12, 13, 14, 15, 16	1,036	−1.38	−1.99	−0.77	−4.45	< .001
	Not Asia	5	1, 3, 5, 7, 8	465	−1.51	−2.87	−0.16	−2.18	.029
IRB	No	2	6, 12	135	−4.73	−8.02	−1.45	−2.83	.005
	Yes	15	1, 2, 3, 4, 5, 7, 8, 9, 10, 11, 12, 13, 14, 15, 16	1,366	−0.99	−1.47	−0.52	−4.09	< .001
Fund	No	5	5, 7, 8, 11, 13	318	−1.67	−2.76	−0.59	−3.03	.002
	Yes	11	1, 2, 3, 4, 6, 9, 10, 12, 14, 15, 16	1,183	−1.31	−1.97	−0.64	−3.86	< .001
Number of participants	< 100	11	5, 6, 7, 8, 9, 11, 12, 13, 14, 15, 16	677	−1.90	−2.68	−1.13	−4.82	< .001
	≥ 100	5	1, 2, 3, 4, 10	824	−0.49	−1.18	0.20	−1.39	.164
Type of pain	Normal delivery	7	5, 6, 7, 9, 11, 12, 14	409	−1.92	−2.93	−0.92	−3.74	< .001
	Cesarian section	4	4, 10, 13, 15	407	−0.96	−1.51	−0.40	−3.38	.001
	Episiotomy	2	1, 2	463	0.24	−0.30	0.78	0.87	.385
	The others	3	3, 8, 16	222	−2.31	−4.44	−0.19	−2.14	.033
Intervention type	Acupuncture	3	1, 3, 8	377	−2.06	−4.30	0.18	−1.80	.072
	Acupressure	13	2, 4, 5, 6, 7, 9, 10, 11, 12, 13, 14, 15, 16	1,124	−1.28	−1.81	−0.75	−4.72	< .001
Intervention tool	Seed	7	2, 6, 9, 12, 13, 14, 16	639	−1.76	−2.79	−0.74	−3.39	.001
	Needle	3	1, 3, 8	377	−2.06	−4.30	0.18	−1.80	.072
	The others	5	4, 5, 7, 10, 15	405	−0.89	−1.39	−0.40	−3.52	< .001
	Not reported	1	11	80	−0.67	−1.12	−0.22	−2.90	.004
Intervention number of stimulus points	< 5	10	1, 2, 3, 4, 5, 6, 7, 13, 14, 16	1,035	−0.87	−1.42	−0.31	−3.07	.002
	≥ 5	6	8, 9, 10, 11, 12, 15	466	−2.24	−3.23	−1.26	−4.45	< .001
Control group intervention	No intervention & routine obstetric care	12	3, 4, 5, 6, 7, 8, 9, 10, 11, 12, 14, 15	896	−1.86	−2.52	−1.20	−5.51	< .001
	Placebo and drug use	4	1, 2, 13, 16	605	−0.16	−0.74	0.42	−0.54	.589
Intervention duration	Not reported or 1 day	10	1, 5, 6, 7, 9, 10, 11, 12, 14, 15	803	−1.53	−2.35	−0.71	−3.67	< .001
	≥ 2 days	6	2, 3, 4, 8, 13, 16	698	−1.24	−2.04	−0.43	−3.00	.003
Intervention session	Not reported or < 2 sessions	10	1, 2, 4, 5, 6, 7, 9, 13, 14, 15	994	−1.05	−1.65	−0.45	−3.43	.001
	≥ 2 sessions	6	3, 8, 10, 11, 12, 16	507	−1.97	−2.97	−0.97	−3.85	< .001
Intervention time/session	Not reported or < 75 seconds	12	2, 3, 5, 6, 7, 8, 9, 10, 11, 13, 14, 16	1,014	−1.56	−2.18	−0.94	−4.92	< .001
	≥ 75 seconds	4	1, 4, 12, 15	487	−0.97	−2.25	0.30	−1.49	.136
Outcome measurement time	Immediately	14	1, 2, 3, 4, 5, 6, 7, 9, 10, 11, 13, 14, 15, 16	1,357	−0.99	−1.46	−0.52	−4.14	< .001
	Delayed	2	8, 12	144	−4.39	−6.99	−1.80	−3.32	.001
Quality score	< 10	5	11, 12, 13, 15, 16	372	−1.20	−2.02	−0.38	−2.87	.004
	≥ 10	11	1, 2, 3, 4, 5, 6, 7, 8, 9, 10, 14	1,129	−1.53	−2.23	−0.82	−4.23	< .001

K= Number of analysis set; N = number of participants; ES = Effect size; CI = Confidence interval; IRB = Institutional review board.

**Table 3.** Meta-Regression Analysis Evaluating Ear Acupressure on Obstetric and Gynecological Pain

Covariates (Ref .)	Estimate	SE	95% CI		Z	*p*
			Lower limit	Upper limit		
Year (< 2019)	−0.11	0.01	−0.13	−0.09	−10.29	< .001
Country (Asia)	0.38	0.12	0.14	0.62	3.11	.002
IRB (No)	3.18	0.30	2.59	3.77	10.52	< .001
Fund (No)	0.53	0.14	0.25	0.81	3.73	< .001
Participants (< 100)	0.99	0.12	0.76	1.22	8.54	< .001
Type of pain						
Normal delivery (Not normal delivery)	−0.91	0.13	−1.18	−0.65	−6.81	< .001
Cesarian section (Not Cesarian section)	−0.35	0.12	−0.60	−0.11	−2.83	.005
Episiotomy (Not episiotomy)	1.32	0.12	1.09	1.55	11.29	< .001
The others (Not the others)	−0.67	0.17	−1.01	−0.33	−3.91	< .001
Intervention type (Acupuncture)	−0.51	0.13	−0.77	−0.26	−3.90	< .001
Intervention tools						
Needle (Not needle)	0.51	0.13	0.26	0.77	3.90	< .001
Seed (Not seed)	−0.12	0.11	−0.35	0.10	−1.06	.290
The others (Not the others)	−0.31	0.12	−0.55	−0.06	−2.46	.014
Intervention stimulus points (< 5)	−1.31	0.13	−1.57	−1.06	−10.07	< .001
Control group intervention (No intervention & routine obstetric care)	1.21	0.11	0.99	1.43	10.68	< .001
Intervention duration (Not reported or < 2 days)	0.25	0.11	0.03	0.47	2.23	.026
Intervention session (Not reported or < 2 sessions)	−1.03	0.12	−1.28	−0.79	−8.29	< .001
Intervention time/session (Not reported or < 75 seconds)	0.55	0.12	0.32	0.79	4.64	< .001
Outcome measurement time (Immediately)	−3.22	0.29	−3.79	−2.65	−11.03	< .001
Quality score (< 10)	0.56	0.13	0.31	0.82	4.27	< .001

Ref. = Reference; SE = Standard error; CI = Confidence interval; IRB = Institutional review board.

## Data Availability

The data that support the findings of this study are available from the corresponding author upon reasonable request.
