# Supplementary material for: The effects of ear acupressure therapy on obstetric and gynecological pain in women: a systematic review and meta-analysis
Source: J Korean Biol Nurs Sci. 2024 Nov 26;26(4):271–87. doi: 10.7586/jkbns.24.035 (PMC13268326; doi:10.7586/jkbns.24.035)
Supplement: Supplementary file 1 [file jkbns-24-035-Appendix-1.pdf]

Appendix 1. Quality Assessment of the Included Studies

Study ID	Author (yr)	Joanna briggs institute of critical appraisal tools checklist for checklist for randomized controlled trials													Total score
		1	2	3	4	5	6	7	8	9	10	11	12	13	
1	Kindberg et al. (2009)	1	0	1	0	0	1	1	1	1	1	1	1	1	10
2	Kwan & Li (2014)	1	0	1	0	0	1	1	1	1	1	1	1	1	10
3	Vas et al. (2019)	1	1	1	1	0	1	0	1	1	1	1	1	1	11
4	Rakchanok et al. (2022)	1	1	1	1	0	1	0	1	1	0	1	1	1	10
5	Mafeton et al. (2018)	1	1	1	1	0	1	1	1	1	1	1	1	1	12
6	Zhu et al. (2023)	1	1	1	1	0	1	1	1	1	1	1	1	1	12
7	Mafetoni & Shimo (2016)	1	1	1	1	0	1	1	1	1	1	1	1	1	12
8	Korelo et al. (2022)	1	0	1	0	0	1	1	1	1	1	1	1	1	10
9	Alimoradi et al. (2020)	1	1	1	1	0	1	1	1	1	1	1	1	1	12
10	Marznaki et al. (2023)	1	1	1	1	0	1	1	1	1	1	1	1	1	12
11	Abedi et al. (2017)	0	1	1	0	0	1	0	1	1	1	1	1	0	8
12	Valiani et al. (2018)	1	0	1	0	0	1	0	1	1	0	1	0	0	6
13	Maryam et al. (2020)	1	0	1	0	0	1	0	1	1	1	1	1	1	9
14	Hu et al. (2024)	1	1	1	0	0	1	1	1	1	1	1	1	1	11
15	Mousavi et al. (2023)	1	0	1	0	0	1	0	1	1	1	1	1	1	9
16	Han et al. (2024)	1	0	1	0	0	1	0	1	1	1	1	1	1	9
Total		15	9	16	7	0	16	9	16	16	14	16	15	14	10.19 ± 1.72

https://doi.org/10.7586/jkbns.24.035
