# Supplementary material for: The effects of ear acupressure therapy on obstetric and gynecological pain in women: a systematic review and meta-analysis
Source: J Korean Biol Nurs Sci. 2024 Nov 26;26(4):271–87. doi: 10.7586/jkbns.24.035 (PMC13268326; doi:10.7586/jkbns.24.035)
Supplement: Supplementary file 2 [file jkbns-24-035-Appendix-2.pdf]

Appendix 2. Studies Included in the Systematic Review and Meta-Analysis

1. Kindberg S, Klünder L, Strøm J, Henriksen TB. Ear acupuncture or local anaesthetics as pain relief during postpartum surgical repair: a randomised controlled trial. BJOG: An International Journal of Obstetrics and Gynaecology. 2009;116(4):569-576. https://doi.org/10.1111/j.1471-0528.2008.02016.x
2. Kwan WS, Li WW. Effect of ear acupressure on acute postpartum perineal pain: a randomised controlled study. Journal of Clinical Nursing. 2014;23(7-8):1153-1164. https://doi.org/10.1111/jocn.12281
3. Vas J, Cintado MC, Aranda-Regules JM, Aguilar I, Rivas Ruiz F. Effect of ear acupuncture on pregnancy-related pain in the lower back and posterior pelvic girdle: a multicenter randomized clinical trial. Acta Obstetricia et Gynecologica Scandinavica. 2019;98(10):1307-1317. https://doi.org/10.1111/aogs.13635
4. Rakchanok T, Komsun S, Paradi S, Supapen L, Junya P, Kornkarn B. Effectiveness of auricular point acupressure with magnetic plate for pain management in acute postpartum cesarean section patients in Thammasat University Hospital: a randomized clinical controlled trial. Journal of Traditional Chinese Medicine. 2022;42(4):611-616. https://doi.org/10.19852/j.cnki.jtcm.2022.04.006
5. Mafetoni RR, Rodrigues MH, Jacob LMdS, Shimo AKK. Effectiveness of auriculotherapy on anxiety during labor: a randomized clinical trial. Revista Latino-Americana de Enfermagem. 2018;26:e3030. https://doi.org/10.1590/1518-8345.2471.3030
6. Zhu Y, Hu Q, Wang J, Li Y, Zhang J, Chang C, et al. Effects of auricular point sticking on labor pain and anxiety. Journal of Acupuncture and Tuina Science. 2023;21(6):460-469. https://doi.org/10.1007/s11726-023-1405-1
7. Mafetoni RR, Shimo AKK. Effects of auriculotherapy on labour pain: a randomized clinical trial. Revista da Escola de Enfermagem da USP. 2016;50(05):726-732. https://doi.org/10.1590/S0080-623420160000600003
8. Korelo RIG, Moreira NB, de Carvalho Miguel BA, da Cruz CdG, de Souza NSP, de Macedo RMB, et al. Effects of auriculotherapy on treatment of women with premenstrual syndrome symptoms: a randomized, placebo-controlled clinical trial. Complementary Therapies in Medicine. 2022;66:102816. https://doi.org/10.1016/j.ctim.2022.102816
9. Alimoradi Z, Kazemi F, Gorji M, Valiani M. Effects of ear and body acupressure on labor pain and duration of labor active phase: a randomized controlled trial. Complementary Therapies in Medicine. 2020;51:102413. https://doi.org/10.1016/j.ctim.2020.102413
10. Marznaki ZH, Hosseinnataj A, Oleson T, Griffiths MD, Alimoradi Z. The effect of auricular acupressure on short-term postoperative pain intensity after cesarean section: a three-arm randomized controlled trial. European Journal of Integrative Medicine. 2023;57:102206. https://doi.org/10.1016/j.eujim.2022.102206
11. Abedi P, Rastegar H, Valiani M, Saadati N. The effect of auriculotherapy on labor pain, length of active phase and episiotomy rate among reproductive aged women. Journal of Family and Reproductive Health. 2017;11(4):185-190.
12. Valiani M, Azimi M, Dehnavi ZM, Mohammadi S, Pirhadi M. The effect of auriculotherapy on the severity and duration of labor pain. Journal of Education and Health Promotion. 2018;7(1):101. https://doi.org/10.4103/jehp.jehp_141_17
13. Maryam A, Fariba A, Azita M, Babak B, Tabandeh S. The effects of auriculotherapy on shoulder pain after a cesarean section. Journal of Acupuncture and Meridian Studies. 2020;13(5):157-162. https://doi.org/10.1016/j.jams.2020.09.002
14. Hu QT, Li Y, Zhu Y, Wang J, Li Q. Effects of multi-mechanism complementary therapy on pain and anxiety during labor latency in primiparous women. Journal of Holistic Nursing. 2024:08980101241232443. https://doi.org/10.1177/08980101241232443
15. Mousavi FS, Golmakani N, Valiani M, Taghanaki HRB, Rezaei F. Comparison of two methods of complementary medicine on postoperative pain and anxiety: a randomized clinical trial. Journal of Education and Health Promotion. 2023;12(1):458. https://doi.org/10.4103/jehp.jehp_1246_22
16. Han S, Kim B, Park H. Auricular acupressure on breast pain among breastfeeding mothers receiving gentle hand techniques: a randomized, single-blind, sham-controlled trial. Journal of Human Lactation. 2024;40(2):248-258. https://doi.org/10.1177/08903344241228545
