# Supplementary material for: The effects of ear acupressure therapy on obstetric and gynecological pain in women: a systematic review and meta-analysis
Source: J Korean Biol Nurs Sci. 2024 Nov 26;26(4):271–87. doi: 10.7586/jkbns.24.035 (PMC13268326; doi:10.7586/jkbns.24.035)
Supplement: Supplementary file 3 [file jkbns-24-035-Appendix-3.pdf]

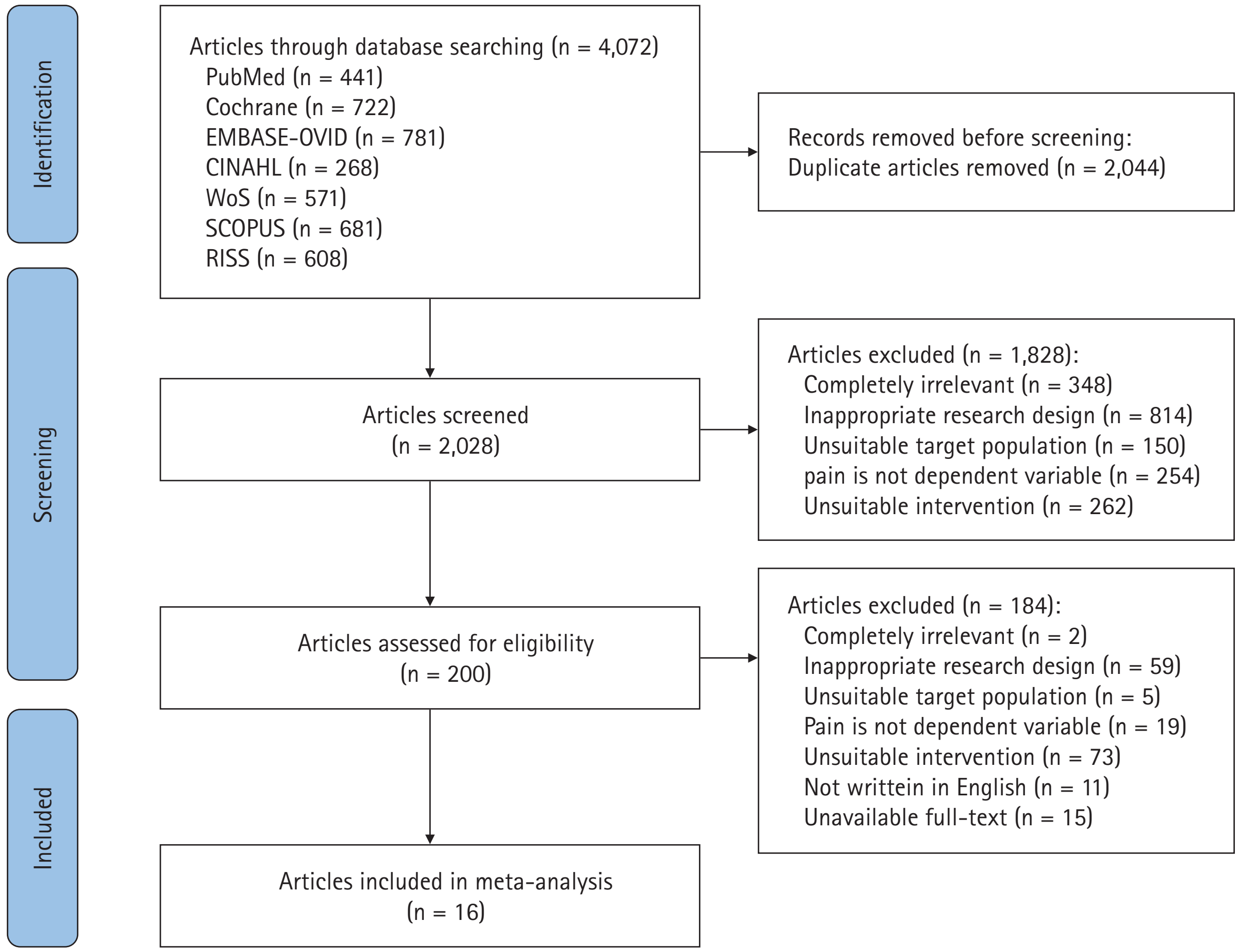

Appendix 3. Preferred reporting items for systematic reviews and meta-analyses flow diagram. RISS = Research Information Sharing Service.
