# Supplementary material for: The effects of ear acupressure therapy on obstetric and gynecological pain in women: a systematic review and meta-analysis
Source: J Korean Biol Nurs Sci. 2024 Nov 26;26(4):271–87. doi: 10.7586/jkbns.24.035 (PMC13268326; doi:10.7586/jkbns.24.035)
Supplement: Supplementary file 4 [file jkbns-24-035-Appendix-4.pdf]

JKBNS

Appendix 4. Exclusion Sensitivity Test of Ear Acupressure Therapy on Obstetric and Gynecological Pain among Women

Author (yr)	K	Hedge's g	95% CI		Z	*p*
			Lower limit	Upper limit		
Kindberg et al. (2009)	15	−1.54	−2.09	−0.99	−5.51	< .001
Kwan & Li (2014)	15	−1.53	−2.13	−0.92	−4.92	< .001
Vas et al. (2019)	15	−1.43	−2.02	−0.84	−4.77	< .001
Rakchanok et al. (2022)	15	−1.50	−2.11	−0.90	−4.90	< .001
Mafeton et al. (2018)	15	−1.45	−2.04	−0.87	−4.86	< .001
Zhu et al. (2023)	15	−1.15	−1.66	−0.64	−4.40	< .001
Mafetoni & Shimo (2016)	15	−1.46	−2.03	−0.89	−4.99	< .001
Korelo et al. (2022)	15	−1.15	−1.66	−0.65	−4.46	< .001
Alimoradi et al. (2020)	15	−1.38	−1.94	−0.81	−4.76	< .001
Marznaki et al. (2023)	15	−1.41	−1.99	−0.83	−4.76	< .001
Abedi et al. (2017)	15	−1.47	−2.06	−0.88	−4.88	< .001
Valiani et al. (2018)	15	−1.28	−1.81	−0.75	−4.73	< .001
Maryam et al. (2020)	15	−1.45	−2.04	−0.86	−4.81	< .001
Hu et al. (2024)	15	−1.47	−2.06	−0.89	−4.95	< .001
Mousavi et al. (2023)	15	−1.44	−2.02	−0.85	−4.83	< .001
Han et al. (2024)	15	−1.50	−2.08	−0.92	−5.04	< .001

K = Number of analysis set; CI = Confidence interval.

https://doi.org/10.7586/jkbns.24.035
