# Supplementary material for: The effects of ear acupressure therapy on obstetric and gynecological pain in women: a systematic review and meta-analysis
Source: J Korean Biol Nurs Sci. 2024 Nov 26;26(4):271–87. doi: 10.7586/jkbns.24.035 (PMC13268326; doi:10.7586/jkbns.24.035)
Supplement: Supplementary file 5 [file jkbns-24-035-Appendix-5.pdf]

Appendix 5. Publication Bias Test of Ear Acupressure Therapy on Obstetric and Gynecological Pain among Women

Begg's test	Tau b	K	S (P-Q)	Ties	Z	*p*
Standard	−0.42	16	−50	0	−2.25	.024
Corrected	−0.41	16	−50	0	−2.21	.027

Egger's regression test	Coef	SE	95% CI		Z	*p*
			Lower limit	Upper limit		
Intercept	−9.63	1.88	−13.30	−5.95	−5.13	< .001
Slope	1.37	0.42	0.54	2.19	3.23	.001

Trim and fill method		95% CI			Z	*p*
		Hedge's g	Lower limit	Upper limit		
Original		−1.41	−1.96	−0.86	−5.01	< .001
Trim and fill correction		−0.25	−0.35	−0.15		

K = Number of analysis set; Coef = Coefficient; SE = Standard error; CI = Confidence interval.
